# Long-term expansion of primary equine keratinocytes that maintain the ability to differentiate into stratified epidermis

**DOI:** 10.1186/s13287-018-0918-x

**Published:** 2018-07-04

**Authors:** Faris Alkhilaiwi, Liqing Wang, Dan Zhou, Terje Raudsepp, Sharmila Ghosh, Siddartha Paul, Nancy Palechor-Ceron, Sabine Brandt, Jennifer Luff, Xuefeng Liu, Richard Schlegel, Hang Yuan

**Affiliations:** 1 Department of Pathology, Georgetown University Medical School, Washington, DC 20057 USA; 2 Department of Oncology, Georgetown University Medical School, Washington, DC 20057 USA; 3 Department of Biochemistry and Molecular Biology, Georgetown University Medical School, Washington, DC 20057 USA; 4 College of Pharmacy, King Abdul Aziz University, Jeddah, Saudi Arabia; 5 Department of Veterinary Integrative Biosciences, Texas A&M University, College Station, TX USA; 6 Equine Clinic, VetOMICs Core Facility, Veterinary University Vienna, Vienna, Austria; 7 Department of Population Health and Pathobiology, College of Veterinary Medicine, North Carolina State University, Raleigh, NC USA

**Keywords:** Equine primary keratinocytes, Three-dimensional cultures, Skin regeneration, Conditional reprogramming

## Abstract

**Background:**

Skin injuries in horses frequently lead to chronic wounds that lack a keratinocyte cover essential for healing. The limited proliferation of equine keratinocytes using current protocols has limited their use for regenerative medicine. Previously, equine induced pluripotent stem cells (eiPSCs) have been produced, and eiPSCs could be differentiated into equine keratinocytes suitable for stem cell-based skin constructs. However, the procedure is technically challenging and time-consuming. The present study was designed to evaluate whether conditional reprogramming (CR) could expand primary equine keratinocytes rapidly in an undifferentiated state but retain their ability to differentiate normally and form stratified epithelium.

**Methods:**

Conditional reprogramming was used to isolate and propagate two equine keratinocyte cultures. PCR and FISH were employed to evaluate the equine origin of the cells and karyotyping to perform a chromosomal count. FACS analysis and immunofluorescence were used to determine the purity of equine keratinocytes and their proliferative state. Three-dimensional air-liquid interphase method was used to test the ability of cells to differentiate and form stratified squamous epithelium.

**Results:**

Conditional reprogramming was an efficient method to isolate and propagate two equine keratinocyte cultures. Cells were propagated at the rate of 2.39 days/doubling for more than 40 population doublings. A feeder-free culture method was also developed for long-term expansion. Rock-inhibitor is critical for both feeder and feeder-free conditions and for maintaining the proliferating cells in a stem-like state. PCR and FISH validated equine-specific markers in the cultures. Karyotyping showed normal equine 64, XY chromosomes. FACS using pan-cytokeratin antibodies showed a pure population of keratinocytes. When ROCK inhibitor was withdrawn and the cells were transferred to a three-dimensional air-liquid culture, they formed a well-differentiated stratified squamous epithelium, which was positive for terminal differentiation markers.

**Conclusions:**

Our results prove that conditional reprogramming is the first method that allows for the rapid and continued in vitro propagation of primary equine keratinocytes. These unlimited supplies of autologous cells could be used to generate transplants without the risk of immune rejection. This offers the opportunity for treating recalcitrant horse wounds using autologous transplantation.

**Electronic supplementary material:**

The online version of this article (10.1186/s13287-018-0918-x) contains supplementary material, which is available to authorized users.

## Background

For thousands of years humans have depended on horses (*Equus caballus*) for transportation in different places of the world [1]. As of 2013, there were more than 60 million horses registered globally, and almost ten millions in the United States with a tremendous economic value [2]. Epidermal tumors such as squamous cell carcinomas as well as other epidermis diseases such as *pemphigus foliaceus* and seborrhea are identified regularly in horses [3, 4]. Furthermore, wound healing in horses is a very complicated process due to the vigorous granulation tissue formed [5]. Therefore, horse skin injuries often lead to the development of chronic non-healing wounds that lack a keratinocyte cover, essential for healing. The pathophysiology of delayed healing in horse wounds has been poorly studied, but the transforming growth factor-beta (TGF-β) expression changes may contribute [6]. While several treatments have been developed for speeding wound healing and inhibiting hypergranulation tissue in horses, the majority of these are of unverified efficacy [7, 8]. In vivo equine wound healing studies and experiments are traumatic and costly for horses [9]. Therefore, a feasible, convenient, and effective in vitro equine keratinocyte model is needed. Optimally, the model would allow for the investigation of the wound pathophysiology and be applicable to skin transplantation.

Stem cell therapy is being increasingly used in horses [10]. For example, mesenchymal stem cells (MSCs) have been used to treat tendon injuries in horses. However, these cells have shown to mediate their effect as trophic cells rather than differentiated cells [11, 12]. Recently, equine induced pluripotent stem cells (eiPSCs) have been generated from equine fibroblasts by overexpressing reprogramming factors Klf4, Sox2, Oct4, and c-Myc by using either transposons or a retroviral vector method [13–15]. The generated eiPSCs have provided a valuable source for researchers to generate different types of somatic cells. More relevant, equine iPSCs could be differentiated into equine keratinocytes (eiPSC-KC) with characteristics suitable for stem cell-based skin constructs to repair damaged or lost skin [16].

It has been difficult to propagate equine keratinocytes in vitro long term using conventional two-dimensional (2D) culture systems [2, 17, 18]. Recently, a new cell culture technique, conditional reprogramming (CR), was developed to efficiently and rapidly establish patient-derived cell cultures from both diseased and normal cells, including tumor cells [19, 20]. With this technique, approximately 1 million epithelial cells can be generated within 7 days [20]. Additionally, these epithelial cells can be propagated indefinitely in vitro, yet maintain the capability to become fully differentiated when transferred into conditions that mimic their biological environment [21, 22].

**Fig. 1:**
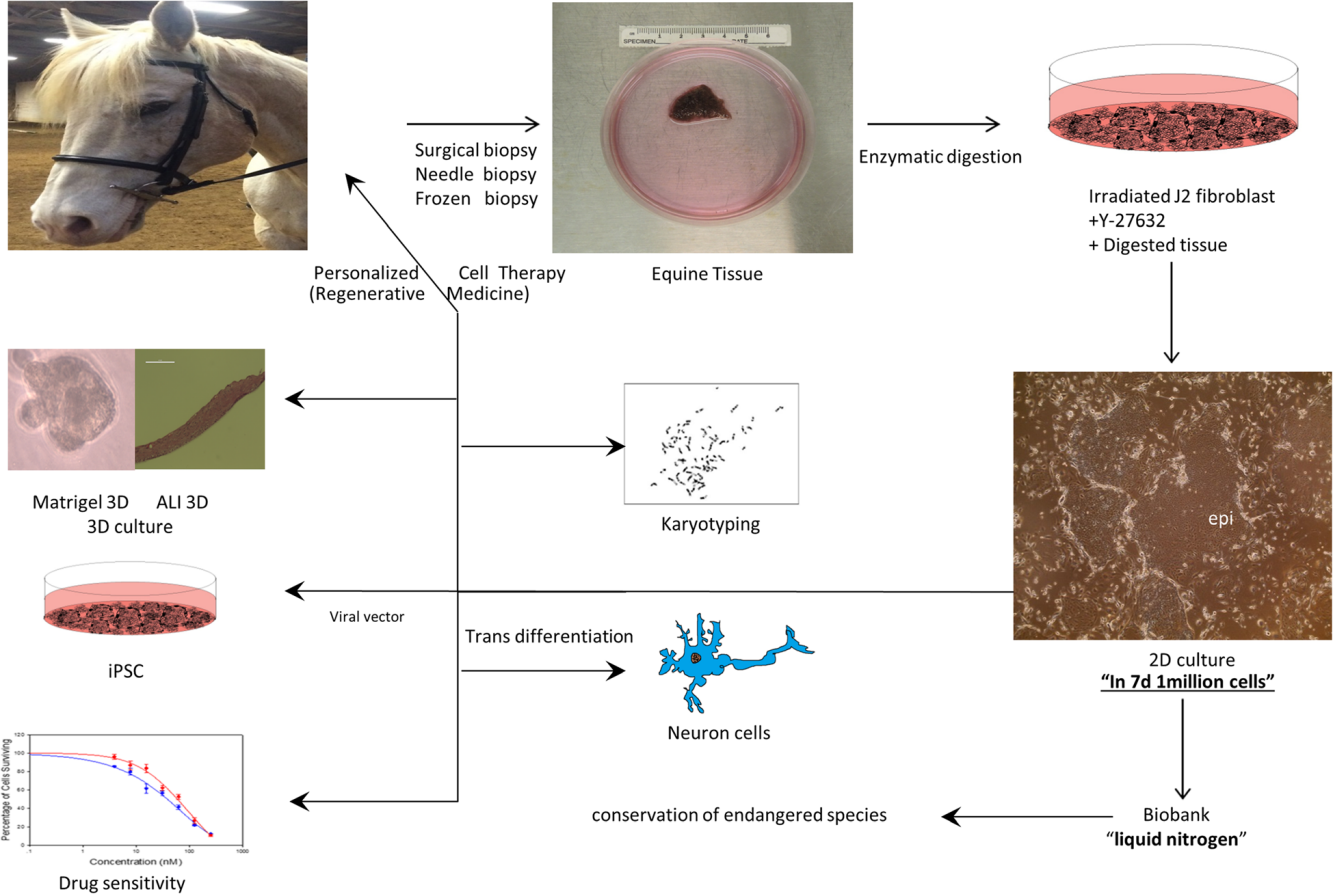
Outline of the CR method for establishing equine keratinocytes cultures and potential uses of generated cells. Equine tissues were obtained from different horses; tissues were digested enzymatically followed by initiation of cell growth and propagation using irradiated mouse fibroblast and ROCK inhibitor. Cells were cryopreserved in liquid nitrogen. The generated equine keratinocytes can be used in numerous applications, including - but not limited to - induced reprogramming stem cells generation (iPSC), transdifferentiation into somatic cells, drug screening, and regenerative medicine

The objective of our study was to establish primary equine keratinocytes using the CR method and to evaluate their potential for transplantation. We demonstrated that the equine cells were rapidly reprogrammed and acquired characteristics of adult stem cells. During this process, the cells became less differentiated and begin to divide rapidly. More importantly, when removed from CR conditions, these cells reverted to their normal differentiated state and organized into structures similar to stratified epithelium from which they were derived. Thus these cultures appear to have potential for regenerative medicine. The equine CR cultures may also have applications to generate iPSC, for drug screening, and transdifferntation into different somatic cells (Fig. 1).

## Methods

### Equine cell harvesting and culturing

Primary horse epidermal keratinocytes and fibroblasts were isolated from two different normal horse tissues (named 1,547, 100) using the CR method as described and used previously [19, 20]. Keratinocytes were cultured under different conditions: (i) in co-culture with irradiated 3 T3 fibroblasts (J2 strain) in F media with or without 10 uM Y-27632 Rho-kinase inhibitor (Enzo Life Sciences, Farmingdale, NY, USA); (ii) in F media with or without 10 uM Y-27632 with no J2; (iii) in CnT-09 media supplemented with A and B supplements (CELLnTEC, Bern, Switzerland) with or without 10 uM Y-27632; (iv) in keratinocyte serum-free medium (KSFM) supplemented with bovine pituitary extract (BPE) and epidermal growth factor 1–53 (EGF 1–53) (KSFM; Invitrogen, Carlsbad, CA, USA) with or without 10 uM Y-27632.

Equine fibroblast cells were purchased from ATCC and cultured in Eagle’s minimum essential medium (EMEM) as recommended, primary equine fibroblasts EF-1547 and EF-100 were grown in complete Dulbecco’s modified Eagle medium DMEM (supplemented with 10% penicillin-streptomycin, 10% L-glutamine and 10% fetal bovine serum). All cells were incubated in 37 °C with 5% CO_2_ incubator. 4.0 × 10^3^ cells/cm^2^ were seeded per passage and split when a confluence approximately 80% was reached. Time in days versus population doubling were plotted to generate the growth curve. All growth conditions are summarized in Table 1. All samples were collected with the owner’s written consent.

**Table 1:** Culture conditions were used for growing equine keratinocytes

Growth media	Calcium concentration^a^ (mM)	Y-27632 concentration (uM)	FBS concentration	Survival	Ability to passage
KSFM+ ^suppa^	0.1	No	No serum	No	No
KSFM+ ^suppa^	0.1	Yes	No serum	No	No
CnT-09+ ^suppb^	1.8	No	10%	yes	No
CnT-09+ ^suppb^	1.8	Yes	10%	Yes	Yes
Fmedia	1.36	No	7.40%	Yes	Yes (1 passage)
Fmedia	1.36	Yes	7.40%	Yes	Yes
Fmedia + irradiated feeders	1.36	Yes	7.40%	Yes	Yes

*suppa* Epidermal growth factor 1–53 (EGF 1–53) and bovine pituitary extract (BPE)

*suppb* Supplements A and Supplements B (200 mM L-glutamine)

^a^Calcium concentration in FBS is not included

### DNA isolation and polymerase chain reaction (PCR) amplification

DNA was isolated from human foreskin keratinocyte, dog cells (k9), 3 T3 murine fibroblasts (J2 strain), equine keratinocytes (EK-100/EK-1547) and equine fibroblasts (EF-100/EF-1547) using DNeasy kit (Qiagen, Germantown, MD, USA). PCR condition for amplification of ATPase6/ATPase8 gene using sense CTATCCGACACACCCAGAAGTAAAG and antisense GATGCTGGGAAATATGATGATCAGA primers was described before [23]. Amplification of human housekeeping gene GAPDH by using sense TCCCTGCCTCTACTGGCGCTGCCAAGGCTG and antisense TCCTTGGAGGCCATGTGGGCCATGAGGTCC primers followed the published method [24].

### Karyotyping

Monolayer primary cultures of equine keratinocyte lines EK1546 and EK100 were grown in CNTY or FY media in T75 cell culture flasks. Semi-confluent (~ 70%) cultures with many mitotic cells were harvested with colcemid (Sigma-Aldrich, St. Louis, MO, USA) following standard procedures [25]. The cells were treated with Optimal Hypotonic solution (Rainbow Scientific, Windsor, CT, USA) following the manufacturer’s protocol, and fixed in methanol:acetic acid (3:1). Chromosome preparations were made on precleaned wet glass slides and stained with Giemsa for initial counting. The sex chromosomes were identified by CBG-banding [26], and refined chromosome analysis and karyotyping were done by GTG-banding [27]. A minimum of 10 cells were captured and analyzed for each technique using an Axioplan2 microscope (Zeiss, Oberkochen, Germany) and Ikaros (MetaSystems GmbH, Altlussheim, Germany) software.

### Fluorescence in situ hybridization (FISH)

Additional verification that the primary keratinocyte cultures EK1547 and EK100 were of pure equine origin and not mixed with other cell lines was done by FISH. We used horse bacterial artificial chromosome (BAC) clones (CHORI-241: http://bacpac.chori.org/equine241.htm) containing select chromosome-specific markers (Table 2). BAC DNA was isolated by Plasmid Midiprep kit (Qiagen), labeled with biotin-16-dUTP or digoxigenin-11-dUTP using Biotin- or DIG-Nick Translation Mix (Roche, Basel, Switzerland), and hybridized to metaphase chromosomes. Hybridizations and signal detection were carried out according to standard protocol [25]. The results were examined with Zeiss Axioplan2 fluorescence microscope and at least ten images were captured and analyzed for each experiment using Isis V5.2 (MetaSystems GmbH) software.

**Table 2:** Horse chromosome specific BAC clones used for FISH

BAC ID	Horse chromosome	Known gene content	Reference	Label and detection
049H16	9	LCORL	Staiger et al. 2016 [35]	Biotin-FITC
076H13	29	CREM	Ghosh et al. 2014 [36]	Digoxigenin-Rhodamine

### Flow cytometry

3 × 10^**5**^ cells were permeabilized with 0.1% v/v Triton X-100 after fixing with 2% PFA.0.5% BSA solution was used for blocking for 30 mins. EK-1547, HFK, and J2 cells were stained with Alexa Fluor (R) 488 Conjugate Pan-Keratin (C11) Mouse mAb (Cell Signaling, Danvers, MA, USA no. 4523S,1:100) for overnight at 4 °C followed by washing twice with 1× PBS/.5 BSA then resuspended in 1× PBS for flow cytometric analysis. 30,000 cells were analyzed on an LSRFFortessa (BD, Franklin Lakes, NJ, USA). Cells were gated by light scatter (FSC vs. SSC) to remove debris. Data analysis on population B was done utilizing FCSExpress 5 (DeNovo software, Glendale, CA, USA) and the percentage of cells staining positive was determined by setting a marker on the unstained sample (1% positive) of each cell line.

HFK cells and J2 fibroblast cells were used as positive and negative control to confirm antibody specificity. Analysis of data from three independent cell cultures was conducted by the flow cytometry and cell sorting shared facility at Georgetown University.

### Immunofluorescence staining

Immunofluorescence staining of equine cells on coverslips was performed for CK14 using kits from Vector Laboratories (Burlingame, CA, USA) and Dako (Glostrup, Denmark) according to manufacturer’s instructions. Briefly, slides were treated with 3% hydrogen peroxide and with an avidin/biotin blocking kit (Invitrogen). The slides were exposed to 10% normal horse serum and to a primary antibodies for CK14 (Abcam, Cambridge, MA, USA no. ab7800, 1:300) diluted in Tris-buffered saline with 0.5% Tween (TBST) for 1 h at room temperature. Slides were exposed to a biotin-conjugated rabbit secondary antibody (Vector Laboratories) diluted in an anti-mouse conjugated horseradish peroxidase labeled polymer from Dako (K4001).The CK14 was visualized withTSA-488 (Life Technology, Waltham, MA, USA ref. T20948). Slides were mounted with Pro-Long Antifade with DAPI. TBST was used for washing throughout. Consecutive cells with the omitted primary antibodies were used as negative controls.

### Immunohistochemistry

Hematoxylin and eosin (H&E) staining and immunohistochemical staining of normal human skin, normal horse skin, breast cancer tissue, and insert’s membranes were performed for Filaggrin, Involucrin, and CK-14. Five-micron sections from formalin-fixed paraffin-embedded tissues were deparaffinized with xylenes and rehydrated through a graded alcohol series. Heat-induced epitope retrieval (HIER) was performed by immersing the tissue sections at 98 °C for 20 min in Tris/EDTA (pH 9.0). Immunohistochemical staining was performed using a horseradish peroxidase-labeled polymer from Dako K4001 according to the manufacturer’s instructions. Briefly, slides were treated with 3% hydrogen peroxide and 10% normal goat serum for 10 min each, and exposed to primary antibodies for 1 h at room temperature. Slides were exposed to the appropriate HRP-labeled polymer for 30 min and DAB chromagen (Dako) for 5 min. Slides were counterstained with hematoxylin (Fisher, Harris Modified Hematoxylin), blued in 1% ammonium hydroxide, dehydrated, and mounted with Acrymount. Consecutive sections with the primary antibody omitted were used as negative controls.

Following antibodies were used: anti-cytokeratin (CK) 14 (Abcam no. ab7800, 1:600, **1:5000**), anti-involucrin (Santa Cruz Biotechnology, Dallas, TX, USA sc-28,557, 1:300, 1:600),anti-filaggrin 14 (Abcam no. ab17808, 1:150).

### Three-dimensional (3D) air-liquid-interface culture

0.4 um PCF inserts (EMD Millipore, Billerica MA, USA) were placed in 24-well plates (Falcon, Chapel Hill, NC, USA) and 3 × 10^5^ EK-1547 or HFK cells were seeded inside the insert using 400ul CNT + Y or F + Y medium correspondingly while 600 uL of medium were added outside the insert. On day 3, all medium was aspirated and 400 uL of differentiation media CnT-PR-3D (CELLnTEC) were added inside the insert and 600 uL outside to allow cells to form intercellular adhesion structure. Plates were incubated for additional 16 h overnight at 37 °C 5% CO. On day 4, all media was removed, and inserts were transferred to 60 × 15 mm dishes (Falcon) and 3.2 ml CnT-PR-3D were added outside of the insert to start the airlift culture. Media was changed every 2–3 days. On day 14 inserts were fixed overnight at 4C using 4% (w/v) paraformaldehyde. Membrane inserts were then placed in 70% ethanol and submitted for H&E staining and sectioning.

## Results

### Establishing equine keratinocytes with the CR method

**Fig. 2:**
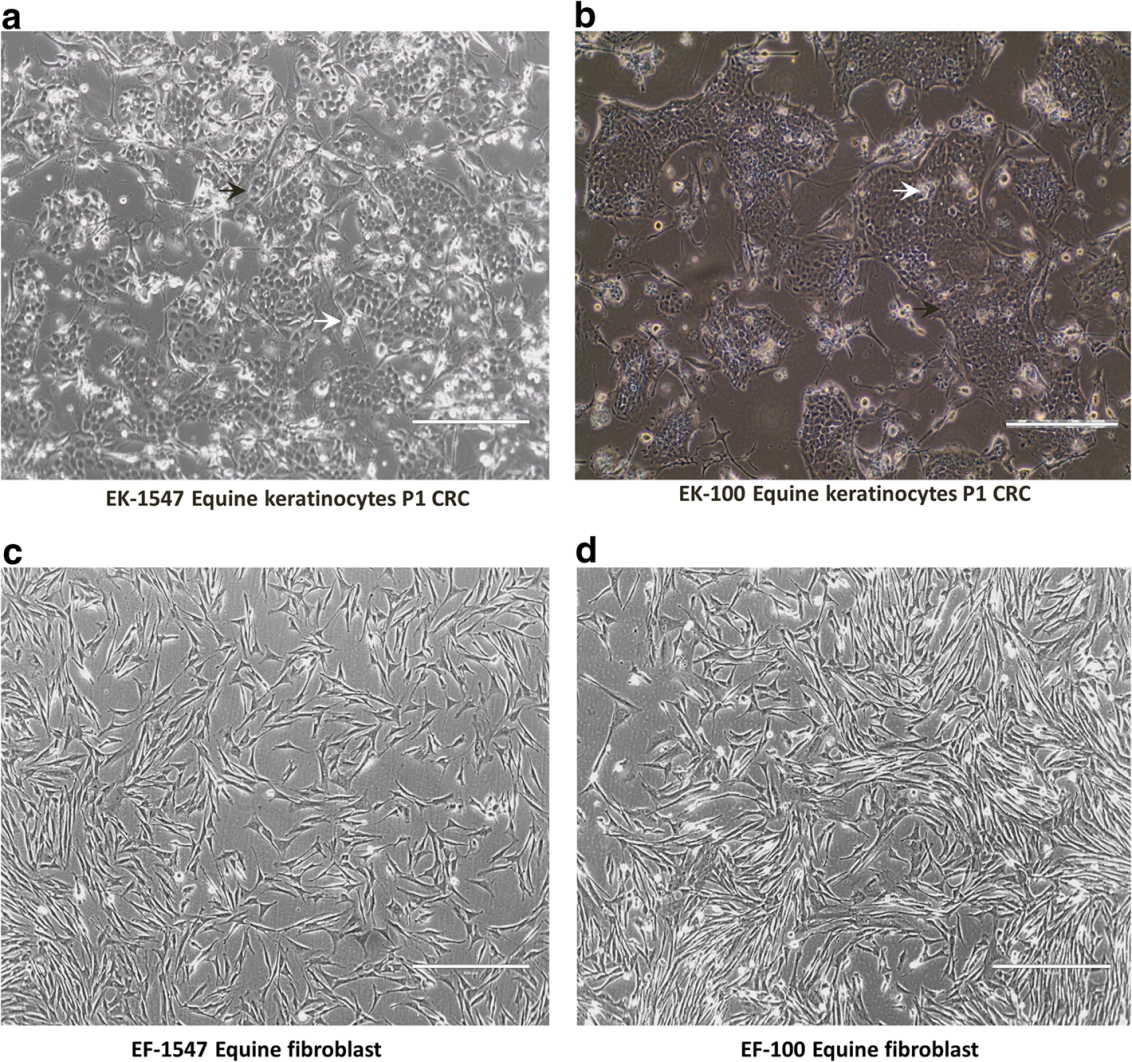
Phase contrast photomicrograph of primary equine keratinocytes and fibroblasts from two different tissues. Cells from horse skin (horse 1) in (**a**) and (**c**) and ones from scrotal skin (horse 2) in (**b**) and (**d**). (**a**) Equine keratinocytes-1547, (**b**) equine keratinocytes-100, (**c**) equine fibroblasts-1547, (**d**) equine fibroblasts-100. (Magnification is ×10 scale bar is 400 μm) *white arrows* indicate irradiated j2 and *black arrows* indicate keratinocytes

Two skin biopsies containing epidermis and dermis were obtained aseptically from two different horses (*Equus caballus*): one strip of scrotal skin was obtained during castration of a Lipizzaner stallion (GUMC-100) and the other one from the neck area of a second horse (GUMC-1547). Approximately 1 × 2 cm biopsies were taken and transferred into DMEM media supplemented with penicillin-streptomycin mix, gentamicin, amphotericin B, and nystatin as described previously [20]. Tissues were transported and stored at 4 °C and processed by incubating them in a mixture of dispase and collagenase to enable physical separation of epithelium and dermal tissue. Later, the epithelium was dispersed into single cells by digestion with (collagenase/trypsin), and the resulting cells were plated on a bed of irradiated Swiss 3 T3 J2 cells (feeder cells) and F medium supplemented with 10 μM Y-27632 (ROCK inhibitor) as previously described [20]. Cobble-stone shaped keratinocyte colonies (Fig. 2a, b black arrows) were readily visible after 2 days, and cultures reached confluence in 5 days. After the first plating, the keratinocytes were seeded at 4.0 × 10^3^ cells/ cm^2^ and passaged every 6–8 days. As reported previously, the irradiated feeders inhibited the outgrowth of fibroblasts in the CR culture conditions [20, 28]. In order to establish fibroblasts from the same equine tissue, some of the single cell suspension was plated in DMEM with 10 μM Y-27532. Spindle-shaped fibroblasts were visible after 2 days (Fig. 2c, d).

### ROCK inhibitor Y-27632 is required for equine keratinocytes proliferation

**Fig. 3:**
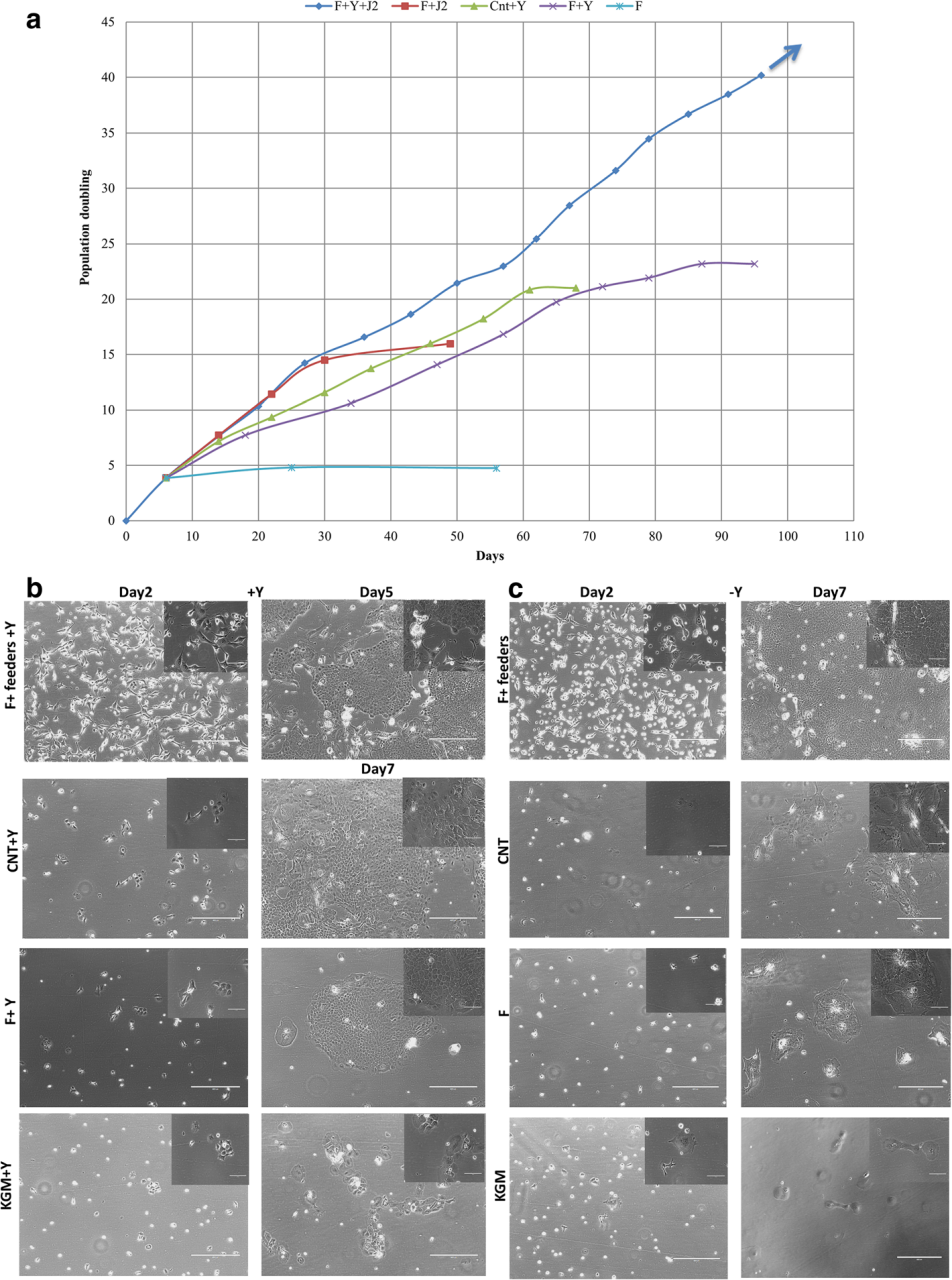
Equine keratinocyte growth was tested in various culture conditions with or without 10 uM Y-27632. (**a**) Growth curve was generated to represent growth rate by plotting population doubling over days in time (graph shows the average of replicates). The *arrow* indicates cells that continued to divide indefinitely. Phase contrast images of primary equine keratinocytes (EK-1547) cultured in co-culture with irradiated fibroblasts, CNT, F, and KSFM with (**b**) or without Y-27632 (**c**). All images were taken on day 2 and day 7 following initial culture without passage (day 5 for feeders+Y condition due to 95% confluency) (×10 magnification. Size bars = 400 μm). *Top right* images show enlarged magnification (×40 magnification, size bars = 100 μm)

To define the optimal in vitro conditions to cultivate and propagate equine keratinocytes, we compared F, KSFM, and CnT09 mediums with or without ROCK inhibitor. We also evaluated F medium with or without feeder cells. The full CR conditions, feeders + F medium + Y-27632, supported equine keratinocyte (EK-1547) growth beyond 40 population doublings (PDs) with an average growth rate of 2.39 days/doubling (Fig. 3a). Without ROCK inhibitor, cell proliferation ceased at 16 population doublings (Fig. 3a). J2 feeder cells were also optimal for cell proliferation since F medium + Y-27632 supported limited cell expansion 25 PDs with a decreased rate of 4.18 days/doubling (Fig. 3a). In this feeder-free system, ROCK inhibitor played a critical role since F medium alone did not support cell growth (Fig. 3a). Equine keratinocytes were not able to proliferate in the synthetic human keratinocyte medium (KSFM) with or without ROCK inhibitor, but rather became rapidly senescent (Fig. 3b, c bottom panel). CnT09, an animal keratinocyte medium, could extend the cell growth to 20 population doublings but only when Y-27632 was added (Fig. 3a). The importance of ROCK inhibitor was also demonstrated with the second equine keratinocytes (EK-100) (Additional file 1: Figure S1). Thus, equine keratinocytes were able to survive and proliferate indefinitely only under complete CR conditions.

### Verification of equine origin and chromosomal stability

**Fig. 4:**
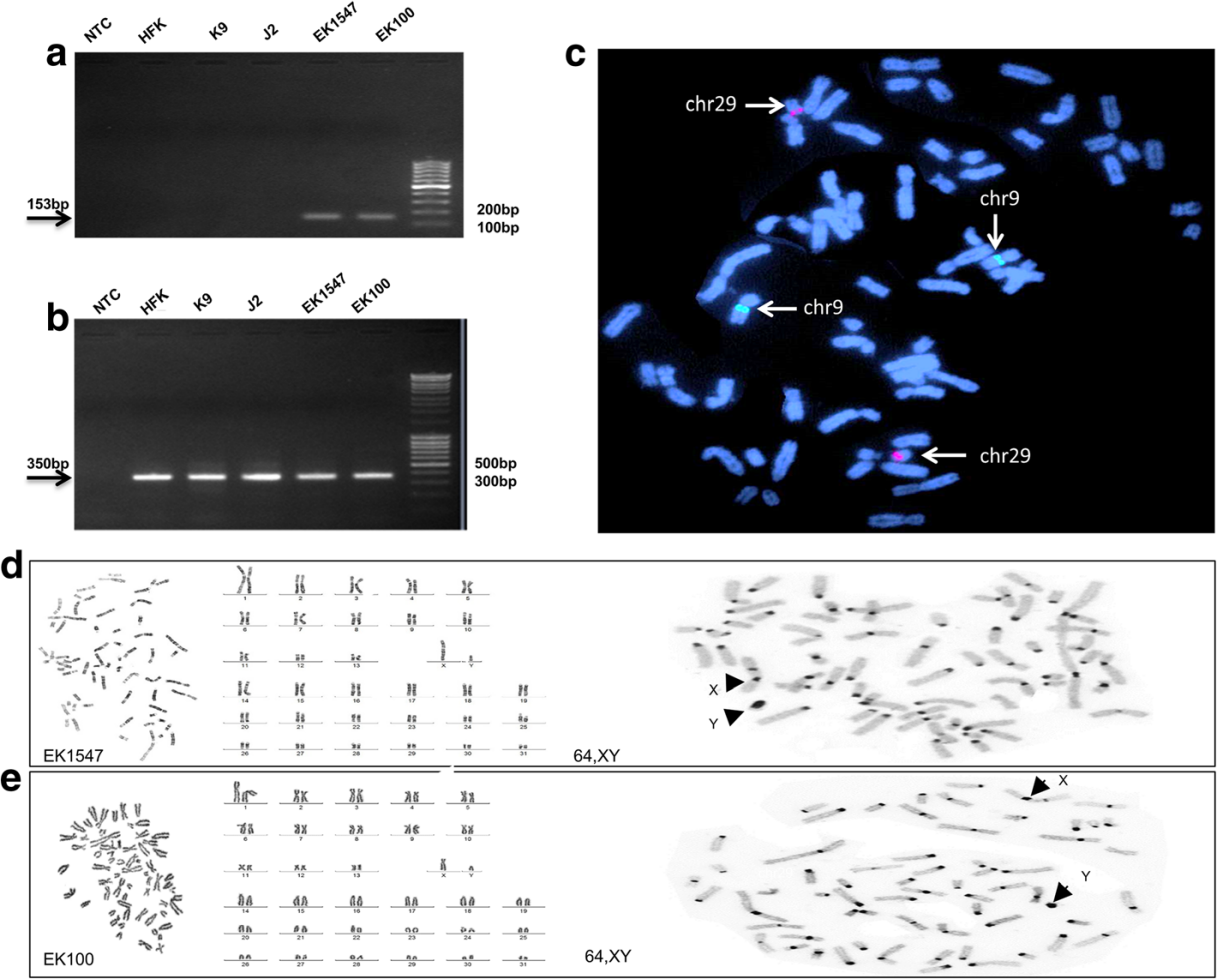
Validation and genotyping of equine keratinocyte cell lines. (**a, b**) PCR amplification with equine MT-ATP6/MT-ATP8 (*top*)and GAPDH (*bottom*) primers; NTC – no template control, HFK - human foreskin keratinocytes, K9 - dog cells, J2 - mouse fibroblasts, EK100 - equine keratinocytes, EK1547- equine keratinocytes; (**c**) FISH with BACs 49H16 (*chr9, green*) and 76H13 (*chr29, red*) in EK1547; (**d**) Cytogenetic analysis of EK1547: G-banded metaphase (*left*), G-banded karyotype (*middle*), C-banded metaphase (*right*); (**e**) Cytogenetic analysis of EK100: Giemsa stained metaphase (*left*), Giemsa stained karyotype (*middle*), C-banded metaphase (*right*)

To verify the equine species identity of the isolated cells, DNAs were extracted from monolayer cultures of the two horse primary keratinocytes lines (EK-1547 and EK-100), as well as from human foreskin keratinocyte (HFK), canine cells (K9) and mouse fibroblasts (J2). Equine-specific mitochondrial ATPase6/ATPase8 gene primers were used to confirm equine origin of the keratinocytes and the expected equine specific band 153 bp was detected only in the two horse primary keratinocyte preparation (EK-1547 and EK-100) (Fig. 4a). Pan-species GAPDH primers were used as loading control (Fig. 4b). Furthermore, we applied FISH with BAC clones specific for select horse autosomes (chrs 9 and 29, Table 2) where two red signals (CREM, chrs 29) and two green signals (LCORL, chrs 9) were detected, which is expected and normal for horse samples (Fig. 4c). Cytogenetic analysis was carried out to corroborate that both cell lines had a normal diploid chromosome number (2n = 64) for the horse, and normal chromosome morphology as revealed by karyotyping Giemsa-stained and G-banded chromosomes (Fig. 4d, e). Both cell lines were confirmed to be genetically male with XY sex chromosomes, as revealed by C-banding (Fig. 4d, e). We did not observe any chromosome abnormalities or any other signs of chromosomal instability, such as breaks, unstained gaps, or abnormal chromosome configurations. These results demonstrate that the cell lines EK1547 and EK100 originate from male horses and carry normal diploid horse karyotypes.

### Pure population of highly proliferative basal keratinocytes

**Fig. 5:**
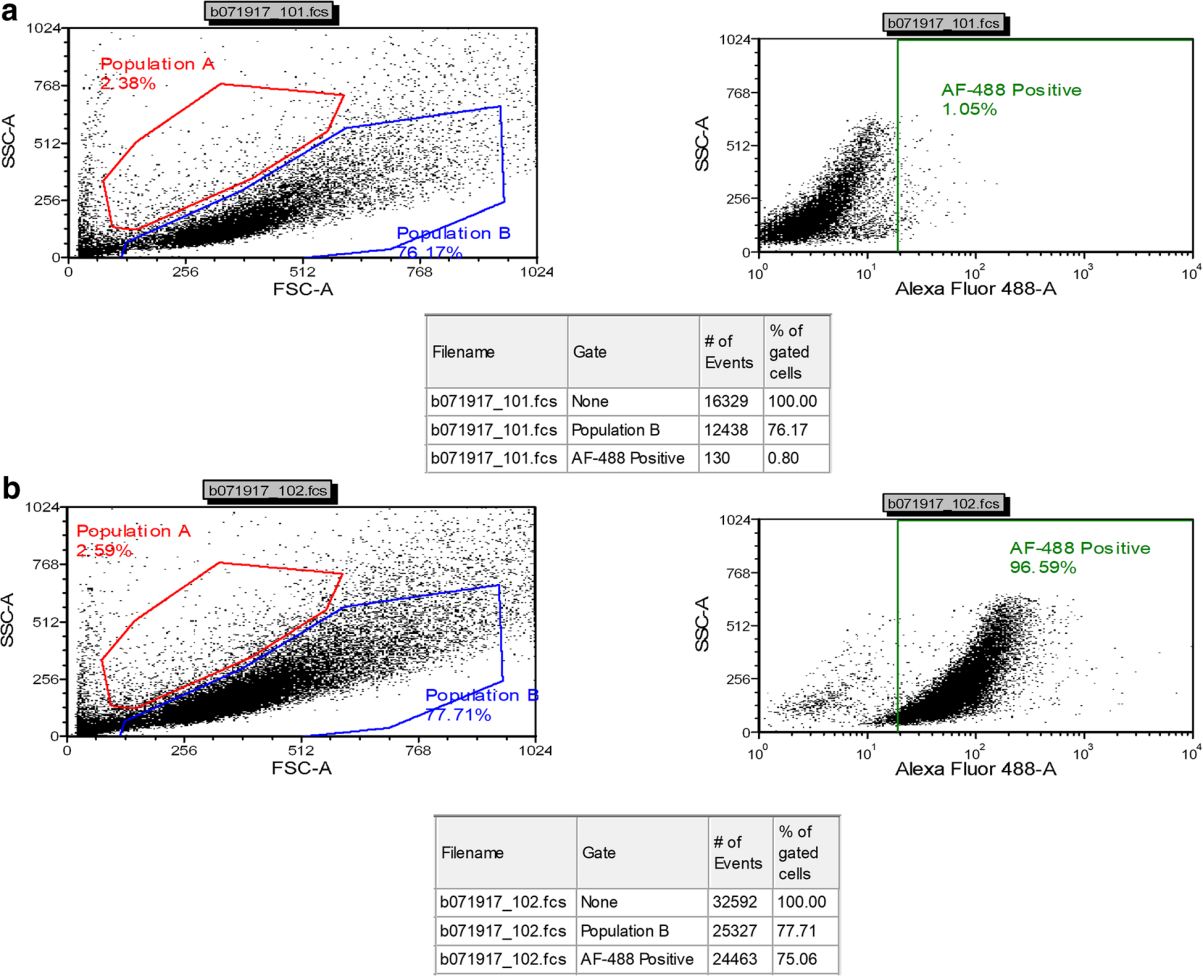
Highly pure population of highly proliferative basal equine keratinocytes. Fluorescence-activated cell sorting (FACS) analysis with pan-cytokeratin antibodies was performed on equine keratinocytes. (**a**) EK-1547 cells without pan-CK antibody as control or (**b**) EK-1547 cells with pan-CK antibody

**Fig. 6:**
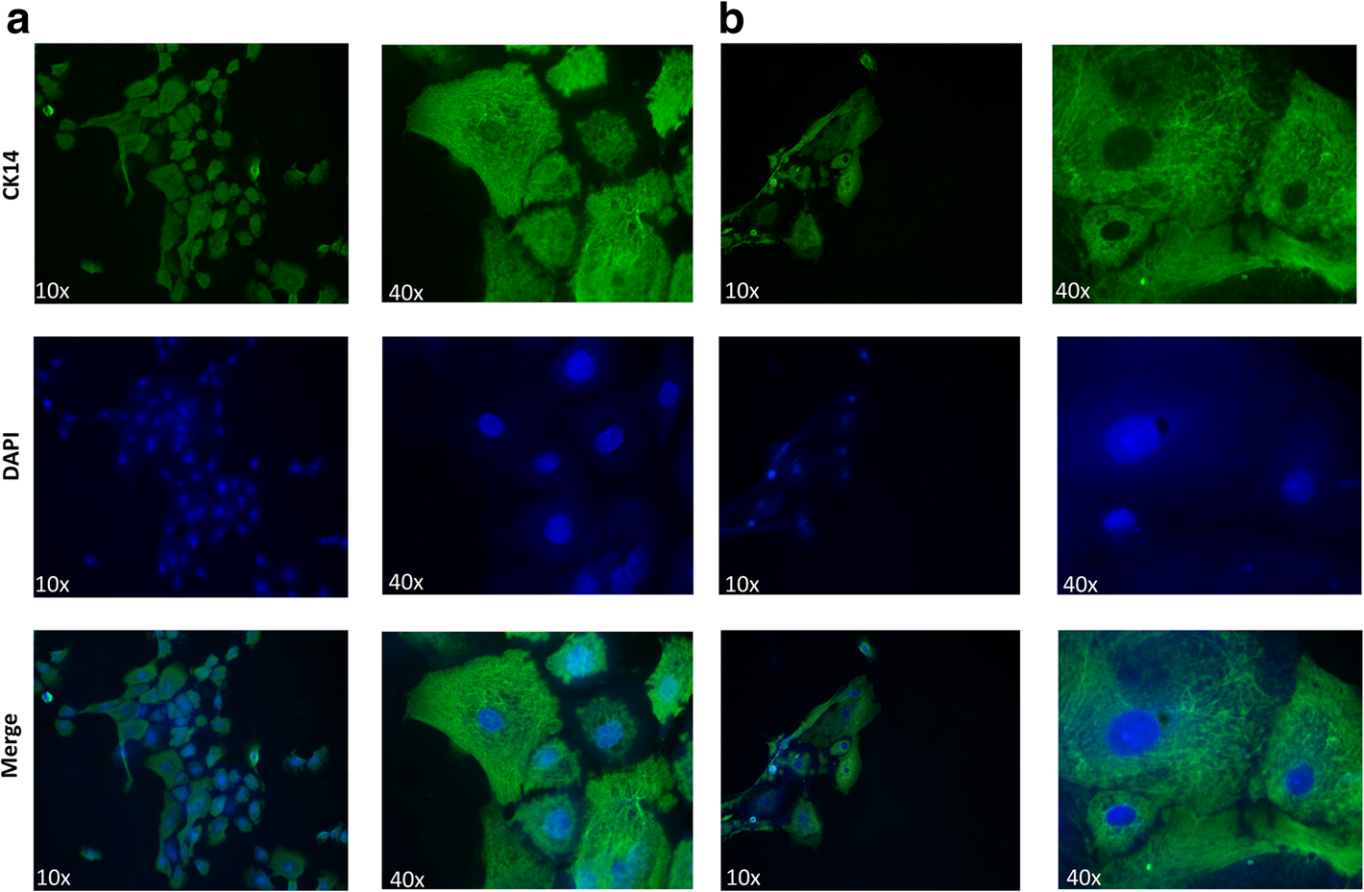
CK-14 Immunofluorescence staining of equine keratinocytes cells. (**a**) EK-1547 cells and (**b**) EK-100 cells were stained for cytokeratin 14, which is localized on the cytoplasm. Fluorescent photomicrographs from three experimental replicates using ×10 and ×40 magnification were captured

When growing keratinocytes from skin tissues, fibroblast contamination represents a common challenge [17]. To determine the purity of the grown keratinocytes, FACS analysis was used. EK-1547 cells at passage 5 were collected and stained with conjugated pan-Keratin antibody. Consistently more than 96% of total cells were positive for pan-Keratin indicating a very high level of keratinocytes (from three independent cell cultures) (Fig. 5a, b). Human foreskin keratinocytes and mouse fibroblasts were used as positive and negative controls respectively (Additional file 1: Figure S2A, B and 2C, D). To confirm the basal nature of the equine keratinocytes, cells were stained for cytokeratin 14 and analyzed using immunofluorescence. The resulting images confirmed that both cell lines EK1547 and EK100 displayed a positive cytoplasmic signal with CK-14 (Fig. 6a, b).

### Equine keratinocytes retain differentiation potential

**Fig. 7:**
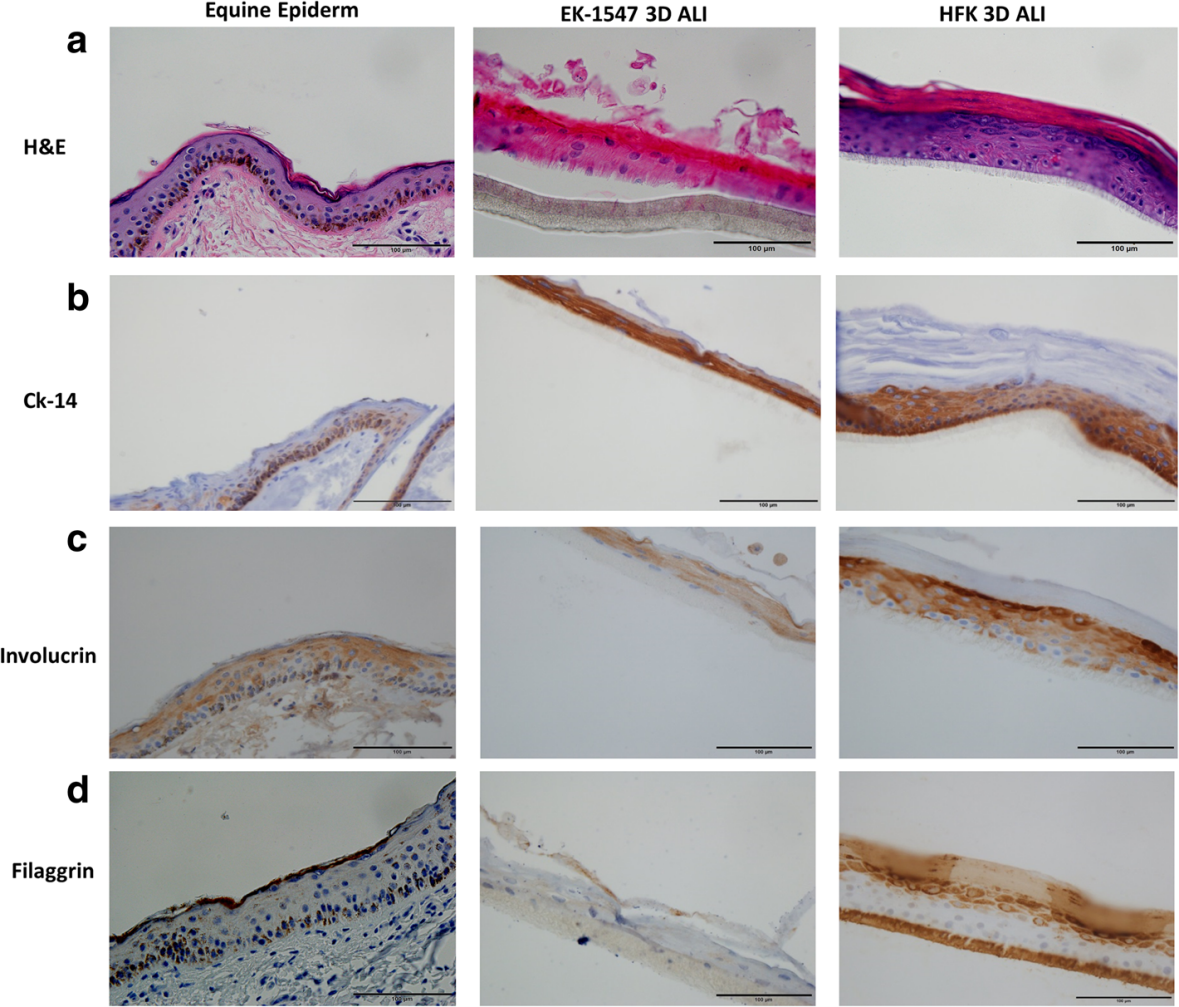
The air-liquid interface of primary equine epithelial cells recapitulate the in vivo skin epithelium. To assess the ability of equine keratinocytes to form a stratified epidermis in ALI (*middle panel*) morphology was compared to normal equine skin (*left panel*) by hematoxylin and eosin (H&E) (**a**) and stratification marker CK14 (**b**) involucrin (**c**) and filaggrin (**d**). HFK in ALI was used as technical control. All images (×40 magnification, scale bar = 100 μm) are representative of three experimental repeats

In order to test whether these proliferating adult stem cell-like equine keratinocytes retained the ability to differentiate, cells were transferred to a 3D air-liquid interface culture in medium lacking Y-27632 [21, 29]. EK-1547 cells were seeded in 0.4 um PCF polycarbonate inserts with 2D medium CnT09 + Y. The cells were incubated at 37 °C with 5% CO2. To induce differentiation, on day 3, medium was replaced with differentiation medium CnT-PR-3D. On day 4, medium on the top of the cells was removed, and cells began to be exposed to air. Hematoxylin and eosin staining of the 3D equine culture (Fig. 7a, 2nd column) revealed that the cells formed an organized differentiated stratified squamous epithelium including a cornified layer similar to equine skin tissue (Fig. 7a, 1st column). Morphological analysis was complemented by immunohistochemistry for the basal cell marker CK14 (Fig. 7b) and the terminal differentiation markers involucrin (Fig. 7c) and filaggrin (Fig. 7d). The 3D human foreskin keratinocytes culture was used as positive control (Fig. 7, 3rd column A-D). Breast cancer tissue and equine skin tissue were used to test the specificity and the reactivity of CK-14 to horse tissue (Additional file 1: Figure S3A, B).

## Discussion

Primary equine keratinocytes have limited growth potential in vitro thereby limiting their use for expansion and regenerative medicine [17]. Despite the high incidence of skin cancer and high rate of granulation in horses’ wounds, the complex interaction of keratinocytes with other cells in the inflamed wound such as neutrophil and fibroblast have not been fully investigated, due to the lack of a reliable in vitro research system [30]. Dahm et al. was the first to isolate and cultivate primary equine keratinocytes from lip epithelium using equine fibroblast feeders or with collagen type I culture coating. However, the equine keratinocytes growth was limited only to a low number of passages, and their growth rate was very slow, needing on average 38 days to reach confluence [17]. Another study showed that equine keratinocytes were isolated from lip epithelium and propagated on collagen type I coated substrate. The cells could be sub-cultured to passage 6 without significant loss of cell character [18]. More recently, isolation of primary equine keratinocytes was accomplished by enzymatic dissociation or with explant culture method [2]. Both methods led to a heterogeneous primary culture comprised of keratinocytes and fibroblasts. There was no evidence of high proliferation rate or long life span of the developed keratinocytes. Using a similar explant culture technique, equine keratinocytes were isolated and propagated using matrigel-coated dishes. These cells were proliferating up to six passages [31]. Using the CR technology, we were able to isolate pure primary equine keratinocyte and efficiently propagate them at the rate of 2.39 days/doubling for 40 PDs.

To the best of our knowledge, this is the first report that shows that primary equine keratinocytes can be propagated indefinitely in an undifferentiated status using an in vitro 2D system. Similarly, we were also able to successfully propagate these cells for long-term using a feeder-free system F medium supplemented with the Rho-Kinase inhibitor Y-27632.

ROCK inhibitor, Y-27632, was first identified being capable of increasing the cloning efficiency of human embryonic stem (ES) cells [32]. Later studies showed that Y-27632 increased the viability of human keratinocyte stem cells [33]. Here, our study shows that the exposure to Y-27632 appears to be required for continued equine keratinocytes proliferation since removal of Y-27632 in any culture condition led to cell senescence. The exact mechanism of Y-27632 on cell immortalization is still unknown. However, it is known that Y-27632 disrupts the actin cytoskeleton, inactivates Rho, and inhibits apoptosis [34]. Interestingly, the effects of Y-27632 are completely reversible, in that equine keratinocytes stopped proliferating and terminally differentiated after its removal when the cells were moved into an air-liquid interphase culture.

Among the earlier studies, it was controversial whether calcium concentration of the medium for equine keratinocytes needed to be low. Dahm et al. showed low concentration of 0.6 mM was required, whereas Visser et al. suggested higher concentration 1.8 mM as optimum concentration [17, 18]. In our culturing system, high calcium concentration 1.8 mM in CNT media or 1.36 mM in F media supported the growth of equine keratinocytes in presence of 10 uM Y-27632. In contrast, KGM with low calcium concentration was not able to support the growth of equine keratinocytes with or without Y-27632. This agrees with earlier studies that skin keratinocytes are able to proliferate at a higher calcium concentration, as long as suitable media and substrate conditions are present [18].

## Conclusions

In our current study, we were able to isolate and propagate pure primary equine keratinocytes rapidly. We also demonstrate that the cells preserved the ability to regenerate a fully stratified epithelium sheet with appropriate spatial and temporal expression of differentiation markers in a short-term in vitro organotypic culture system. This system can be used for autologous skin regeneration therapy for horses, potentially satisfying a huge unmet medical need.

## Additional file

Additional file 1: **Figure S1.** Equine keratinocytes (EK-100) were cultured in various culture conditions. Representative phase contrast images of primary equine keratinocytes (EK-100) cultured in (**a**) co-culture with irradiated fibroblasts+ 10 uM Y-27632, (**b**) F + 10 uM Y-27632 (**c,d**) CNT with or without 10 uM Y-27632 and (**e**)KSFM+ 10 uM. All images were taken on and day7 following initial culture without passage (×10 magnification. Size bars = 400 μm). *Top right* images show enlarged magnification (×40 magnification, size bars = 100 μm). **Figure S2.** Fluorescence-activated cell sorting (FACS) analysis of human keratinocytes (HFK) and mouse fibroblasts (j2) using pan-cytokeratin antibody. HFK cells were incubated without (**a**) pan-CK antibody or (**b**) with pan-CK antibody, (**c**) J2 fibroblasts without pan-CK antibody, or (**d**) with pan-CK antibody. **Figure S3.** Validation of antibodies for equine tissues. Specificity and reactivity of CK-14 was tested by using diluted concentration of CK14 1:600, 1:5000, and no antibody respectively in (**a**) breast cancer tissue and (**b**) equine skin tissue. All images (×40 magnification, scale bar = 100 μm) are representative of three experimental repeats. (PPTX 5023 kb)

## Abbreviations

2D: Two-dimensional
3D: Three-dimensional
CK: Cytokeratin
CR: Conditional reprogramming
DMEM: Dulbecco’s modified Eagle medium
eiPSCs: Equine induced pluripotent stem cells
EK-1547 EK-100: Equine keratinocytes
EMEM: Eagle’s minimum essential medium
FISH: Fluorescent in situ hybridization
HFK: Human foreskin keratinocyte
K9: Canine cells
KSFM: Human keratinocyte medium
MSCs: Mesenchymal stem cells
PCR: Polymerase chain reaction
PDs: Population doublings
ROCK: Rho-associated protein kinase
TGF-β: Transforming growth factor-beta
