# Supplementary material for: Long-term expansion of primary equine keratinocytes that maintain the ability to differentiate into stratified epidermis
Source: Stem Cell Res Ther. 2018 Jul 4;9:181. doi: 10.1186/s13287-018-0918-x (PMC6032561; doi:10.1186/s13287-018-0918-x)
Supplement: Supplementary file 1 — Figure S1. Equine keratinocytes (EK-100) were cultured in various culture conditions. Representative phase contrast images of primary equine keratinocytes (EK-100) cultured in (a) co-culture with irradiated fibroblasts+ 10 uM Y-27632, (b) F + 10 uM Y-27632 (c,d) CNT with or without 10 uM Y-27632 and (e)KSFM+ 10 uM. All images were taken on and day7 following initial culture without passage (×10 magnification. Size bars = 400 μm). Top right images show enlarged magnification (×40 magnification, size bars = 100 μm). Figure S2. Fluorescence-activated cell sorting (FACS) analysis of human keratinocytes (HFK) and mouse fibroblasts (j2) using pan-cytokeratin antibody. HFK cells were incubated without (a) pan-CK antibody or (b) with pan-CK antibody, (c) J2 fibroblasts without pan-CK antibody, or (d) with pan-CK antibody. Figure S3. Validation of antibodies for equine tissues. Specificity and reactivity of CK-14 was tested by using diluted concentration of CK14 1:600, 1:5000, and no antibody respectively in (a) breast cancer tissue and (b) equine skin tissue. All images (×40 magnification, scale bar = 100 μm) are representative of three experimental repeats. (PPTX 5023 kb) [file 13287_2018_918_MOESM1_ESM.pptx]

## Slide 1
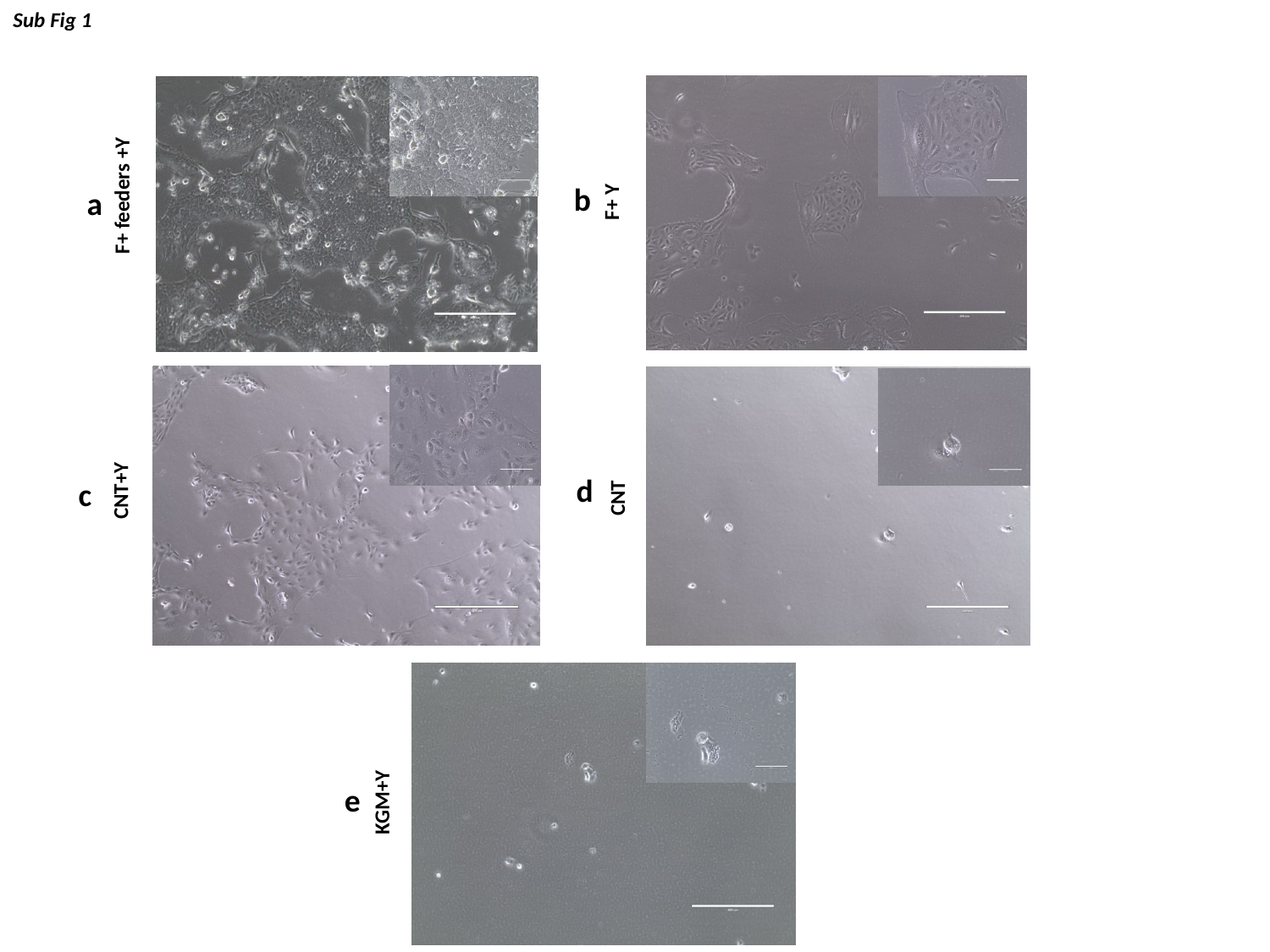

Sub Fig 1
F+ feeders +Y
F+ Y
b
a
CNT+Y
CNT
d
c
KGM+Y
e

## Slide 2
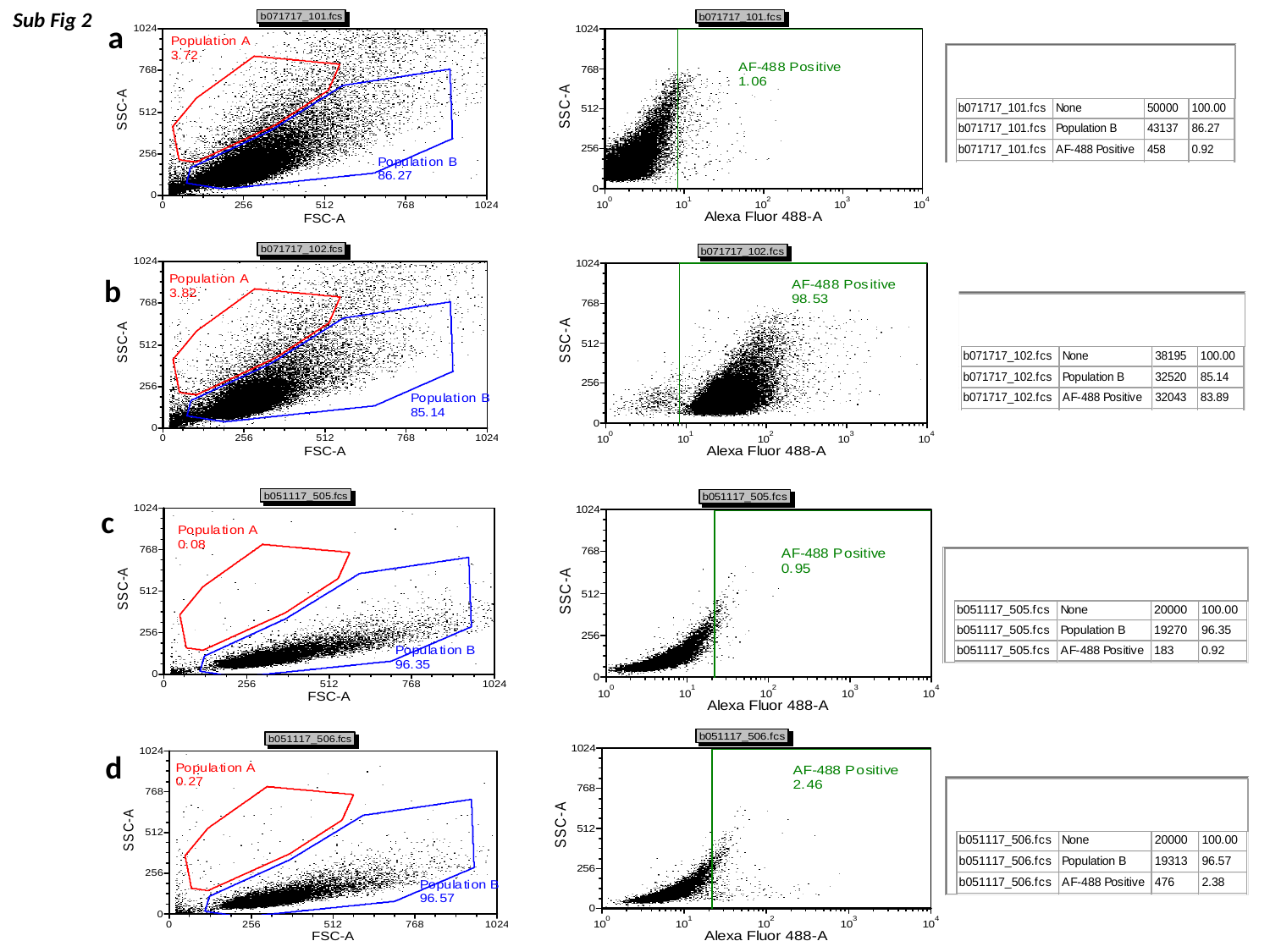

Sub Fig 2
a
b
c
d

## Slide 3
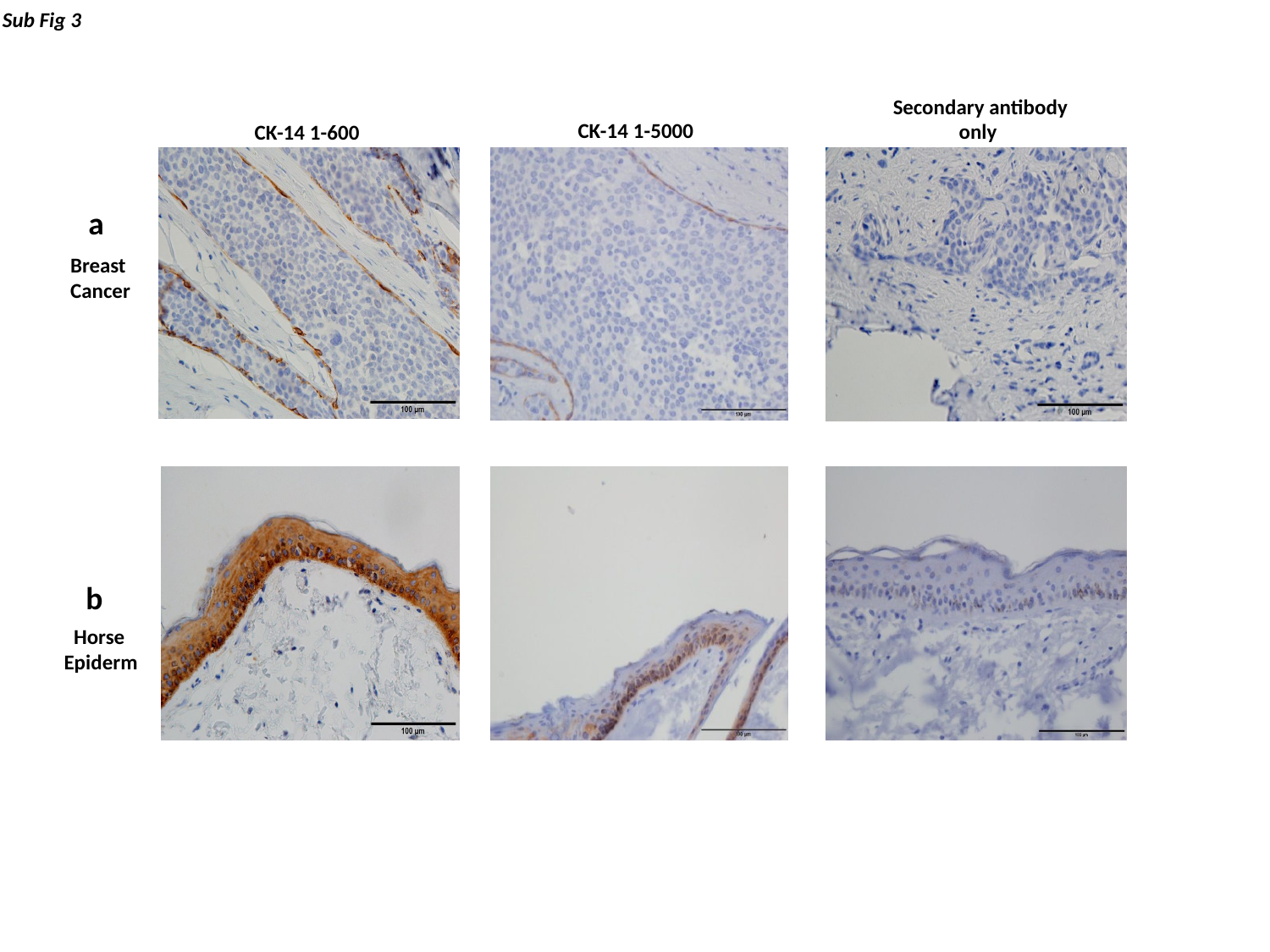

Sub Fig 3
Secondary antibody only
CK-14 1-600
CK-14 1-5000
a
Breast
Cancer
b
 Horse
Epiderm
